# A cross-sectional study of the cost and nutritional content of plant-based meat-imitation products in supermarkets and plant-based products in restaurants in the United Kingdom

**DOI:** 10.1177/02601060251344449

**Published:** 2025-05-27

**Authors:** Grace Monori, Anjum Memon, Gemma Archer

**Affiliations:** 1 12190 Brighton and Sussex Medical School, Brighton, UK; 2 Department of Primary Care and Public Health, 12190 Brighton and Sussex Medical School, Brighton, UK; 3 Department of Medical Education, 12190 Brighton and Sussex Medical School, Brighton, UK

**Keywords:** Meat, fish, poultry, vegan, plant-based, nutrient content, price

## Abstract

**Background:** There is a global trend of increasing consumption of plant-based foods, yet little is known about the nutritional composition and price of plant-based meat alternatives and restaurant items in the United Kingdom, and how they compare to non-plant-based options. **Aim:** This study compared the nutritional content and cost of plant-based and non-plant-based foods/products sold in UK supermarkets and restaurants. **Methods:** In this cross-sectional study, meat-based products (*n* = 1228) and plant-based-imitation products (*n* = 147) were identified from five supermarkets. Plant-based (*n* = 54) and equivalent non-plant-based (*n* = 54) items were identified from ten restaurants. Nutritional information (energy, fat, saturated fat, carbohydrate, sugar, fibre, protein, and salt) and price of the products was compared for plant-based and non-plant-based products using the Mann–Whitney *U* test and Wilcoxon Signed Rank test. **Results:** For supermarkets, the plant-based meat-imitation products had lower median saturated fat and protein, and higher median sugar and fibre per 100 g compared with non-plant-based products (all results *p* < 0.001). Plant-based supermarket products were more expensive (median £1.02/100 g vs £0.85/100 g; *p* < 0.001). For restaurant products, plant-based items were significantly lower in energy (*p* < 0.001), saturated fat (*p* = 0.017), sugar (*p* = 0.007) and protein (*p* < 0.001), and higher in fibre (*p* = 0.009), per serving, compared with non-plant-based products. There was no difference between the cost of plant-based and non-plant-based restaurant products per serving (*p* = 0.39). **Conclusion:** Plant-based meat-imitation products available in UK supermarkets were lower in saturated fat, but higher in sugar compared with meat options, and were significantly more expensive. Plant-based products sold in restaurants may provide a healthier alternative, at no additional cost per serving.

## Introduction

### Plant-based and vegan diets

Plant-based diets can be defined as diets excluding foods or ingredients of animal origin, to varying degrees (Hargreaves et al., 2023), with the Physicians Committee for Responsible Medicine defining plant-based diets as one that ‘consists of exclusively plant foods, including fruit, vegetables, grains, and legumes, and avoids meat, dairy, and eggs’ (Physicians Committee for Responsible Medicine, 2025). The complete exclusion of foods of animal origin is synonymous with a vegan diet (Hargreaves et al., 2023; Key et al., 2022). Plant-based and vegan diets are gaining rising popularity worldwide (The Vegan Society, 2024), including in the United Kingdom, where 4% of individuals identify as vegan (Statista, 2024d). There is also a wider trend of declining meat intakes; the United Kingdom average daily meat consumption per person decreased by 17% between 2008 and 2019 (Stewart et al., 2021).

### Plant-based meat alternatives

In line with the rising popularity of plant-based and vegan diets, the global market of plant-based meat substitutes is increasing, with an estimated 65% growth predicted between 2023 and 2028, to $16.8 billion (Statista, 2024a). Plant-based meat-imitation products are foods designed to replicate the sensory qualities of the animal products that they imitate, including visual appearance and taste (Andreani et al., 2023). Plant-based meat alternatives contribute 40% of total energy intake in vegans (Gehring et al., 2021), and represent a convenient and acceptable alternative to meat-containing products (Onwezen et al., 2021; Szenderák et al., 2022). Therefore, it is important to understand how the nutritional composition of plant-based meat alternatives compares with meat products.

In a study of 43 plant-based products in UK supermarkets, Coffey et al. (2023), found that plant-based products were significantly higher in fibre and sugar, and lower in energy, saturated fat and protein, compared to meat products. These findings are consistent with a larger study of over 2000 vegan meat alternatives from supermarkets across five European countries, including the United Kingdom (Petersen and Hirsch, 2023). Further studies of vegetarian plant-based meat alternatives have confirmed these findings in the United Kingdom (Alessandrini et al., 2021), as well as internationally (Costa-Catala et al., 2023; Curtain and Grafenauer, 2019), whilst a study of products available in German supermarkets found some plant-based products had a higher protein content compared with the meat alternatives (Gréa et al., 2023).

Cost is also an important consideration in food purchasing behaviour, particularly for individuals with a low income (Steenhuis et al., 2011). Despite research suggesting that individuals following a plant-based diet spend less on food compared with meat-eaters (Pais et al., 2022), perceived higher cost has been reported as a barrier to adopting a plant-based diet (Rickerby and Green, 2024). Therefore, understanding the relative cost of plant-based options will help to illustrate whether cost is a barrier to consumers making plant-based choices in the United Kingdom (Steenhuis et al., 2011).

To the best of our knowledge, only a single UK-based study (Coffey et al., 2023) has conducted a cost-comparison of plant-based meat alternatives and meat products, and found that meat alternatives were significantly higher in price. No further research has investigated this in a larger sample of products.

### Plant-based options in restaurants

Food purchased from restaurants, fast food outlets and takeaways makes a significant contribution to nutritional intake in the United Kingdom. Foods eaten in restaurants and food outlets contribute to over a fifth of total energy intake in the United Kingdom (Department of Health and Social Care, 2020). As a result of the significant contribution to dietary intakes, an understanding of the nutritional composition of plant-based meals consumed outside the home, and how they compare to non-plant-based alternatives, is of interest.

To the best of our knowledge, only two studies have compared the nutritional composition of exclusively plant-based and non-plant-based restaurant products in UK restaurants (Guess et al., 2023; Kaminski et al., 2024). Both studies found that plant-based products were significantly lower in protein, fat, saturated fat and salt, and higher in carbohydrate, sugar and fibre, compared with non-plant-based options. Results were mixed for energy, with one study showing a similar energy content of plant-based and non-plant-based products (Kaminski et al., 2024), whilst the other reported a significantly lower energy content of plant-based products (Guess et al., 2023). Whilst these studies included a large number of products from several countries, including the United Kingdom, these studies did not compare products matched closely in terms of product type. This may result in comparison of plant-based products that would not be seen by consumers as direct alternatives to their non-plant-based counterparts. Furthermore, to the best of our knowledge, no studies have compared the cost of exclusively plant-based and non-plant-based products in UK restaurants.

### Aims

The aim of this study was to understand how the nutritional composition and cost of meat, fish and poultry products, compare with equivalent plant-based-imitation products, across multiple product categories sold in UK supermarkets. Furthermore, a nutritional and cost comparison of plant-based and non-plant-based UK restaurant menu items was conducted.

## Methods

### Study design

We conducted an observational, cross-sectional study to compare the nutritional content and cost of plant-based and non-plant-based products sold in UK supermarkets and restaurants. This study is reported in keeping with STROBE guidelines (Supplemental Material 2) (Elm et al., 2007).

### Supermarkets

#### Selection of supermarkets

Supermarket chains with the highest UK market share in April 2024 were selected for inclusion (Statista, 2024b). The top five supermarkets by market share were Tesco (27.4% of the market share), Sainsbury's (15.3%), Asda (13.4%), Aldi (10%) and Morrisons (8.7%) (Statista, 2024b). These supermarkets have a total of 74.8% of the UK market share (Statista, 2024b).

#### Product selection

Non-plant-based products across nine product categories were selected for inclusion, with comparable plant-based imitation products selected for each category. For the purpose of this study, plant-based products were defined as those completely free of animal-derived ingredients, and are therefore synonymous with vegan products. The decision was made to exclude plant-based products that contained dairy or eggs to ensure this paper provides a novel insight, as comparisons including vegetarian meat alternatives have been done multiple times previously (Alessandrini et al., 2021; Costa-Catala et al., 2023; Curtain and Grafenauer, 2019; Gréa et al., 2023). Furthermore, vegan products are increasingly relevant as a rising minority follow a vegan diet in the United Kingdom (Statista, 2024d). The categories were: bacon (pork), burgers (beef), chicken (coated), chicken (uncoated), fish (coated), ham (pork), meatballs (beef), mince (beef) and sausages (pork).

Data were collected for supermarket products between 13 May and 2 June 2024. Products were identified by searching through relevant product categories on supermarket websites (e.g., all chilled vegetarian and vegan products) (Aldi Stores Limited, 2024; Asda, 2024; J Sainsbury's plc, 2024; Tesco, 2024; Wm Morrison Supermarkets Limited, 2024). Additionally, the search function on supermarket websites was used, with search terms indicated in Supplemental Table 1.

If a product was available in multiple pack sizes, then all sizes were included in the analysis, to account for price variations. A detailed summary of the inclusion and exclusion criteria for products is shown in Supplemental Material 1 and Supplemental Table 2.

### Restaurants

#### Selection of restaurants

The ten restaurant chains with the highest popularity rating in the United Kingdom were selected for inclusion: Burger King, Costa Coffee, Domino's Pizza, Greggs, J D Wetherspoon, KFC, McDonald's, Pizza Express, Pizza Hut, and Subway (Statista, 2024c). These chains include both dine-in and take-away food outlets. To mitigate the potential variation in prices of products between restaurants in different locations, the branch of each restaurant brand closest to central London (WC2N 5HF) was selected for inclusion. All restaurants were within 0.7 miles of this location.

#### Product selection

Data were collected for restaurant products between 27 May and 18 June 2024. The specific menu for each store location was accessed through the restaurants’ websites (Burger King Corporation, 2024; Domino's Pizza UK and Ireland Limited, 2024b; Greggs plc, 2024; J D Wetherspoon plc, 2024b; KFC, 2024b; Pizza Express, 2024a; Pizza Hut UK Ltd, 2024b), or if this was not available, then through their app (Costa, 2024a) or a third-party food ordering company, Just Eat (Just Eat, 2024a, 2024b). The menu for each restaurant was searched for items labelled as vegan or plant-based. All available menus for each restaurant were screened, e.g., breakfast menu, main menu. Drinks and children's menu items were excluded.

For each plant-based menu item, a comparable non-plant-based item was sought (including vegetarian and meat-based options). Only if a comparable non-plant-based item was available, then both the plant-based and non-plant-based item were eligible for inclusion. Plant-based and non-plant-based menu items were considered comparable if they had a similar structure (e.g., salad, curry, cookie), item size, and number of servings per menu item.

### Data collected

For all supermarket products, cost data were collected from supermarket websites (Aldi Stores Limited, 2024; Asda, 2024; J Sainsbury's plc, 2024; Tesco, 2024; Wm Morrison Supermarkets Limited, 2024). Nutritional information was collected from supermarket websites or from the product website if not available from the supermarket. If the same product was available across multiple retailers, then the cheapest price was recorded.

For restaurants, cost data were collected from restaurants’ websites (Burger King Corporation, 2024; Domino's Pizza UK and Ireland Limited, 2024b; Greggs plc, 2024; J D Wetherspoon plc, 2024b; KFC, 2024b; Pizza Express, 2024a; Pizza Hut UK Ltd, 2024b), or if this was not available, through their app (Costa, 2024a), and if this was not available then from Just Eat's website (Just Eat, 2024a, 2024b). Nutritional information for restaurant products was collected from the restaurants’ websites (Burger King Corporation, 2024; Costa, 2024b; Domino's Pizza UK and Ireland Limited, 2024a; Greggs plc, 2024; J D Wetherspoon plc, 2024a; KFC, 2024a; McDonald's, 2024; Pizza Express, 2024b; Pizza Hut UK Ltd, 2024a; Subway, 2024).

The following information was collected for all products: weight (grams), standard retail price (Great British pounds (GBP)), energy (calories), fat (grams), saturated fat (grams), carbohydrate (grams), sugar (grams), fibre (grams), protein (grams) and salt (grams). The aforementioned nutrient levels were recorded per 100 g of product for supermarket items, and both per menu item serving and per 100 g for restaurant items.

If sodium content was reported, it was converted to salt content by multiplying by 2.5. If a nutrient quantity was reported as less than a cut-off (e.g., < 0.5 g), as this is accepted to equate to a negligible amount (Department of Health, 2016), a value of zero was recorded. For supermarket products, if nutritional information was only available for 100 g of a cooked product, it was converted to the nutritional content per 100 g of uncooked product, using standard conversion factors for weight changes during cooking (available in Supplemental Table 3) (Food Standards Agency, 2010). The cost per 100 g product, per 100 calories and per 100 g of protein were calculated.

### Data quality control

Products were reviewed for duplicate items (i.e., the same product by the same manufacturer in the same pack size), which were deleted. The data for products with the highest and lowest value for weight, cost and each nutrient level were cross-checked with the published data for accuracy. Errors were corrected for four products. The accuracy of nutritional information was verified based on a method used in a similar analysis (Guess et al., 2023). The recorded macronutrient contents were multiplied by their Atwater factors (9 kcal/g for fat, 4 kcal/g for carbohydrate and protein, 2 kcal/g for fibre) (Food and Agriculture Organization of the United Nations, 2024), to derive a calculated theoretical energy content. If the calculated energy content was not within ± 10% of the recorded energy content, or the product had missing data for carbohydrate, protein, fat or fibre content, then the accuracy of the data was reviewed. If the nutritional information was recorded correctly from the published data, and (for products with no missing data) the theoretical calculated energy content was not within ± 10% of the recorded energy content, then the products were excluded from the analysis (n = 16 supermarket products, *n* = 1 restaurant items).

### Statistical analysis

Statistical analysis was conducted in IBM SPSS Statistics, version 29.0.1.0 (IBM, 2023). The statistical tests performed are summarised below, with further details included in Supplemental Material 3.

#### Supermarket data

Nutritional content per 100 g of product, and cost per 100 g of product, per 100 calories and per 100 g of protein were summarised using the median and interquartile range (IQR). Each variable was compared for plant-based meat-imitation products and non-plant-based products across each category individually, and across products from all categories combined, using Mann–Whitney *U* test mean ranks, as the data was non-parametric. Two-tailed *p*-values are reported, with the threshold for statistical significance set at a *p*-value of <0.05.

#### Restaurant data

For restaurant products, serving size, nutritional content per serving and per 100 g of product, and cost per 100 g of product, per 100 calories and per 100 g of protein are summarised using the median and IQR. The Wilcoxon Test was used to compare plant-based and non-plant-based restaurant products, as the data was non-parametric but was paired. The Sign test was used to test for a difference between paired data when a symmetrical distribution of the differences between pairs was not satisfied despite transformation of variables. Two-tailed *p*-values are reported, with the threshold for statistical significance set at a *p*-value of <0.05.

## Results

### Supermarket products

#### Product availability

A total of 147 plant-based meat-imitation products and 1228 non-plant-based supermarket products were included in the study across the nine product categories: bacon (plant-based *n* = 6, non-plant-based *n* = 162), burgers (plant-based *n* = 23, non-plant-based *n* = 75), chicken (coated) (plant-based *n* = 36, non-plant-based *n* = 228), chicken (uncoated) (plant-based *n* = 19, non-plant-based *n* = 128), fish (coated) (plant-based *n* = 10, non-plant-based *n* = 191), ham (plant-based *n* = 9, non-plant-based *n* = 195), meatballs (plant-based *n* = 13, non-plant-based *n* = 19), mince (plant-based *n* = 10, non-plant-based *n* = 62), and sausages (plant-based *n* = 21, non-plant-based *n* = 168).

#### Nutrition

Data on the energy, fat, saturated fat, sugar, and salt content were available for all plant-based and non-plant-based products. Carbohydrate content was missing for 5 non-plant-based (0.4% sample) and 2 plant-based (1.4% sample) products, fibre content was missing for 42 non-plant-based (3.4% sample) and 4 plant-based (2.7% sample) products, and protein content was missing for 5 non-plant-based (0.4% sample) and 2 plant-based (1.4% sample) products.

The median and IQR of the nutritional content for plant-based and non-plant-based products, alongside the results of the Mann–Whitney *U* tests are shown in Tables 1 and 2. Further results of the Mann–Whitney *U* test, including mean ranks and *U*-values, are detailed in Supplemental Table 4.

**Table 1.** Median (IQR) of energy, fat, saturated fat, carbohydrate, sugar and fibre content of plant-based and non-plant-based supermarket products in the UK.

	Energy (kcal/100 g)	*p*-value	Fat (g/100 g)	*p*-value	Saturated fat (g/100 g)	*p*-value	Carbohydrate (g/100 g)	*p*-value	Sugar (g/100 g)	*p*-value	Fibre (g/100 g)	*p*-value
Bacon												
Plant-based (*n* = 6)	179.3 (30.0)	0.648	9.8 (5.9)	0.423	**1.1** (**0.8)**	**<0**.**001**	**7.0** (**5.9)**	**<0**.**001**	**1.2** (**0.9)**	**<0**.**001**	**5.2** (**2.8)**	**<0**.**001**
Non-plant-based (*n* = 162)	170.8 (52.4)		11.2 (5.1)		**4.2** (**2.2)**		**0.5** (**0.6)**		**0.0** (**0.4)**		**0.3** (**0.4)**	
Burgers												
Plant-based (*n* = 23)	176.0 (72.9)	0.988	9.9 (7.4)	0.238	**2.2** (**3.3)**	**<0**.**001**	**5.1** (**2.3)**	**<0**.**001**	**0.7** (**1.3)**	**0**.**005**	**4.4** (**2.2)**	**<0**.**001**
Non-plant-based (*n* = 75)	180.0 (37.7)		11.5 (3.8)		**5.1** (**1.7)**		**2.5** (**2.5)**		**0.3** (**0.7)**		**0.4** (**0.6)**	
Chicken, coated												
Plant-based (*n* = 36)	229.1 (74.3)	0.399	12.2 (4.9)	0.060	1.3 (1.1)	0.088	16.5 (7.4)	0.053	0.9 (0.6)	0.331	**5.2** (**2.6)**	**<0**.**001**
Non-plant-based (*n* = 228)	228.3 (43.9)		11.5 (4.3)		1.6 (1.0)		15.6 (6.1)		0.8 (0.6)		**1.2** (**0.7)**	
Chicken, uncoated												
Plant-based (*n* = 19)	**163.6** (**61.0)**	**<0**.**001**	**7.2** (**8.5)**	**<0**.**001**	**0.9** (**0.8)**	**0**.**005**	**3.0** (**3.4)**	**<0**.**001**	**0.2** (**0.5)**	**<0**.**001**	**5.8** (**3.0)**	**<0**.**001**
Non-plant-based (*n* = 128)	**106.0** (**13.4)**		**1.4** (**2.1)**		**0.5** (**0.8)**		**0.0** (**0.0)**		**0.0** (**0.0)**		**0.0** (**0.0)**	
Fish, coated												
Plant-based (*n* = 10)	**214.2** (**34.1)**	**0**.**004**	**11.7** (**4.5)**	**0**.**006**	**1.0** (**1.1)**	**0**.**031**	**24.3** (**6.8)**	**<0**.**001**	**1.0** (**0.8)**	**0**.**004**	**3.1** (**1.0)**	**<0**.**001**
Non-plant-based (*n* = 191)	**193.3** (**35.3)**		**8.6** (**3.5)**		**0.7** (**0.3)**		**16.6** (**4.7)**		**0.7** (**0.4)**		**0.9** (**0.4)**	
Ham												
Plant-based (*n* = 9)	**159.0** (**100.5)**	**0**.**037**	7.4 (9.0)	0.058	0.8 (0.4)	0.061	**3.5** (**5.4)**	**<0**.**001**	0.8 (2.2)	0.963	**4.5** (**2.5)**	**<0**.**001**
Non-plant-based (*n* = 195)	**118.0** (**17.0)**		2.8 (1.6)		1.0 (0.7)		**1.2** (**1.4)**		1.0 (1.5)		**0.5** (**0.7)**	
Meatballs												
Plant-based (*n* = 13)	163.0 (52.6)	0.106	**6.1** (**5.9)**	**0**.**044**	**0.6** (**1.3)**	**<0**.**001**	**6.2** (**3.7)**	**<0**.**001**	**1.0** (**1.1)**	**<0**.**001**	**5.4** (**2.1)**	**<0**.**001**
Non-plant-based (*n* = 19)	182.9 (23.3)		**11.1** (**4.7)**		**5.1** (**2.2)**		**2.3** (**1.5)**		**0.1** (**0.5)**		**0.0** (**0.2)**	
Mince												
Plant-based (*n* = 10)	141.0 (106.4)	0.344	**3.7** (**10.9)**	**0**.**042**	**0.8** (**2.7)**	**<0**.**001**	**6.0** (**5.0)**	**<0**.**001**	**0.7** (**2.1)**	**<0**.**001**	**4.6** (**3.6)**	**<0**.**001**
Non-plant-based (*n* = 62)	168.0 (78.0)		**7.9** (**9.6)**		**3.1** (**4.0)**		**0.0** (**0.2)**		**0.0** (**0.0)**		**0.0** (**0.0)**	
Sausages												
Plant-based (*n* = 21)	**166.0** (**52.3)**	**<0**.**001**	**8.9** (**8.0)**	**<0**.**001**	**1.3** (**3.3)**	**<0**.**001**	6.6 (6.7)	0.061	1.0 (1.2)	0.697	**4.7** (**2.2)**	**<0**.**001**
Non-plant-based (*n* =168)	**233.9** (**47.4)**		**17.8** (**5.5)**		**6.4** (**2.1)**		6.0 (5.7)		1.0 (1.2)		**0.9** (**0.7)**	
All products												
Plant-based (*n* = 147)	188.0 (73.0)	0.102	10.0 (7.1)	0.253	**1.0** (**1.3)**	**<0**.**001**	**7.5** (**10.1)**	**<0**.**001**	**0.8** (**0.8)**	**<0**.**001**	**4.6** (**2.5)**	**<0**.**001**
Non-plant-based (*n* = 1228)	183.0 (89.4)		9.4 (8.6)		**1.8** (**3.7)**		**2.7** (**13.1)**		**0.6** (**1.0)**		**0.6** (**1.0)**	

g: grams; IQR: interquartile range; kcal: kilocalories; *n*: number of products.

Statistically significant results using Mann–Whitney *U* tests are highlighted in bold.

**Table 2.** Median (IQR) of protein and salt content, and cost of plant-based and non-plant-based supermarket products in the UK.

	Protein (g/100 g)	*p*-value	Salt (g/100 g)	*p*-value	Cost (£/100 g)	*p*-value	Cost (£/100 kcal)	*p*-value	Cost (£/100 g protein)	*p*-value
Bacon										
Plant-based (*n* = 6)	**14.5** (**5.3)**	**0**.**045**	2.2 (1.2)	0.516	**2.19** (**1.31)**	**<0**.**001**	**1.31** (**0.62)**	**<0**.**001**	**13.06** (**9.76)**	**<0**.**001**
Non-plant-based (*n* = 162)	**17.4** (**4.7)**		2.3 (0.7)		**1.04** (**0.76)**		**0.54** (**0.46)**		**5.57** (**3.44)**	
Burgers										
Plant-based (*n* = 23)	**14.6** (**3.2)**	**0**.**004**	**0.9** (**0.4)**	**<0**.**001**	**1.10** (**0.46)**	**0**.**001**	**0.58** (**0.19)**	**0**.**001**	**7.68** (**5.70)**	**<0**.**001**
Non-plant-based (*n* = 75)	**15.9** (**2.6)**		**0.6** (**0.2)**		**0.77** (**0.19)**		**0.43** (**0.21)**		**5.05** (**1.82)**	
Chicken, coated										
Plant-based (*n* = 36)	**11.0** (**2.8)**	**<0**.**001**	**1.0** (**0.3)**	**<0**.**001**	**1.10** (**0.50)**	**<0**.**001**	**0.47** (**0.19)**	**<0**.**001**	**9.48** (**5.22)**	**<0**.**001**
Non-plant-based (*n* = 228)	**14.5** (**3.4)**		**0.7** (**0.4)**		**0.83** (**0.47)**		**0.38** (**0.23)**		**5.47** (**2.98)**	
Chicken, uncoated										
Plant-based (*n* = 19)	**17.0** (**8.4)**	**<0**.**001**	**0.8** (**0.8)**	**<0**.**001**	**1.25** (**0.89)**	**<0**.**001**	0.86 (0.70)	0.210	**7.29** (**4.71)**	**<0**.**001**
Non-plant-based (*n* = 128)	**22.5** (**4.0)**		**0.2** (**0.1)**		**0.72** (**0.21)**		0.68 (0.22)		**3.28** (**1.25)**	
Fish, coated										
Plant-based (*n* = 10)	**4.3** (**4.7)**	**<0**.**001**	**0.9** (**0.5)**	**0**.**019**	0.89 (0.45)	0.819	0.41 (0.19)	0.361	**22.21** (**16.89)**	**<0**.**001**
Non-plant-based (*n* = 191)	**12.0** (**1.8)**		**0.7** (**0.3)**		1.00 (0.49)		0.52 (0.26)		**8.77** (**4.36)**	
Ham										
Plant-based (*n* = 9)	19.3 (13.7)	0.272	1.7 (0.5)	0.397	**2.60** (**0.92)**	**<0**.**001**	1.38 (1.54)	0.091	**15.38** (**7.92)**	**<0**.**001**
Non-plant-based (*n* = 195)	21.0 (3.1)		1.6 (0.6)		**1.54** (**0.88)**		1.20 (0.67)		**7.12** (**3.44)**	
Meatballs										
Plant-based (*n* = 13)	**13.0** (**3.1)**	**<0**.**001**	**1.1** (**0.3)**	**<0**.**001**	0.75 (0.64)	0.872	0.44 (0.29)	0.650	6.56 (4.77)	0.136
Non-plant-based (*n* = 19)	**17.5** (**4.3)**		**0.7** (**0.3)**		0.71 (0.40)		0.40 (0.27)		4.54 (2.05)	
Mince										
Plant-based (*n* = 10)	**17.2** (**3.1)**	**<0**.**001**	**0.7** (**0.4)**	**<0**.**001**	0.70 (0.84)	0.566	0.41 (0.24)	0.513	3.77 (4.17)	0.406
Non-plant-based (*n* = 62)	**21.0** (**2.2)**		**0.2** (**0.1)**		0.70 (0.26)		0.49 (0.28)		3.48 (1.27)	
Sausages										
Plant-based (*n* = 21)	11.0 (5.6)	0.092	**1.4** (**0.2)**	**<0**.**001**	**0.83** (**0.32)**	**<0**.**001**	**0.50** (**0.18)**	**<0**.**001**	**8.13** (**3.32)**	**<0**.**001**
Non-plant-based (*n* =168)	12.7 (3.3)		**1.1** (**0.3)**		**0.66** (**0.32)**		**0.28** (**0.14)**		**4.99** (**2.37)**	
All products										
Plant-based (*n* = 147)	**13.0** (**5.6)**	**<0**.**001**	1.0 (0.5)	0.052	**1.02** (**0.74)**	**<0**.**001**	**0.53** (**0.40)**	**0**.**019**	**8.67** (**6.25)**	**<0**.**001**
Non-plant-based (*n* = 1228)	**16.0** (**7.6)**		0.9 (1.1)		**0.85** (**0.55)**		**0.49** (**0.42)**		**5.46** (**3.65)**	

g: grams; IQR: interquartile range; kcal: kilocalories; n: number of products.

Statistically significant results using Mann–Whitney *U* tests are highlighted in bold.

Across all products, there was no significant difference between the energy (median (IQR) plant-based: 188.0(73.0) kcal/100 g, non-plant-based: 183.0(89.4) kcal/100 g, *U* =  82,827.0, *p* = 0.102), fat (plant-based: 10.0(7.1) g/100 g; non-plant-based: 9.4(8.6) g/100 g, *U* =  85,056.5, *p* = 0.253) or salt content (plant-based 1.0(0.5) g/100 g, non-plant-based 0.9(1.1) g/100 g, *U* = 81,419.5, *p* = 0.052) of plant-based and non-plant-based products per 100 g. The saturated fat (plant-based: 1.0(1.3) g/100 g, non-plant-based: 1.8(3.7) g/100 g, *U* =  66,172.0, *p* < 0.001) and protein content (plant-based: 13.0(5.6) g/100 g, non-plant-based: 16.0(7.6) g/100 g, *U* =  56,014.5, *p* < 0.001) were significantly lower in plant-based products compared with non-plant-based products per 100 g. Across all products, the carbohydrate (plant-based: 7.5(10.1) g/100 g, non-plant-based: 2.7(13.1) g/100 g, *U* =  59,116.5, *p* < 0.001), sugar (plant-based: 0.8(0.8) g/100 g; non-plant-based: 0.6(1.0) g/100 g, *U* =  66,703.5, *p* < 0.001), and fibre content (plant-based: 4.6(2.5) g/100 g; non-plant-based: 0.6(1.0) g/100 g; *U* = 2015.5, *p* < 0.001) were significantly higher for plant-based products, compared with non-plant-based products per 100 g.

Whilst the results for fibre were consistent across all product categories, with plant-based products containing significantly more fibre per 100 g compared with non-plant-based products across all product categories (*p* < 0.001), the results for other nutrients were more variable (Tables 1 and 2). For example, the saturated fat content was significantly higher in plant-based uncoated chicken (median (IQR) plant-based: 0.9(0.8) g/100 g, non-plant-based: 0.5(0.8) g/100 g, U = 743.0, *p* = 0.005), and plant-based fish (plant-based: 1.0(1.1) g/100 g; non-plant-based: 0.7(0.3) g/100 g, U = 571.5, *p* = 0.031), compared with their non-plant-based equivalents. Conversely, the saturated fat content was significantly lower in plant-based bacon (plant-based: 1.1(0.8)g/100 g; non-plant-based: 4.2(2.2) g/100 g, *U* = 66.0, *p* < 0.001), plant-based burgers (plant-based: 2.2(3.3) g/100 g; non-plant-based: 5.1(1.7) g/100 g, *U* = 250.5, *p* < 0.001), plant-based meatballs (plant-based: 0.6(1.3) g/100 g; non-plant-based: 5.1(2.2) g/100 g, *U* = 12.0, *p* < 0.001), plant-based mince (plant-based: 0.8(2.7) g/100 g; non-plant-based: 3.1(4.0) g/100 g; *U* = 108.0, *p* < 0.001) and plant-based sausages (plant-based: 1.3(3.3) g/100 g; non-plant-based 6.4(2.1) g/100 g, U = 196.0, *p* < 0.001), compared with their non-plant-based equivalents.

#### Cost

Cost data per 100 g product were available for all plant-based and non-plant-based products, as was cost per 100 calories. Cost per 100 g protein was missing for five non-plant-based (0.4% sample) and 2 plant-based (1.4% sample) products, due to a missing protein content for these products.

The median and IQR of the cost of plant-based and non-plant-based products, alongside the results of the Mann Whitney U tests are shown in Table 2, with mean ranks and U-values detailed in Supplemental Table 5.

Across all products, the cost as expressed in pounds per 100 g product (plant-based: £1.02(0.74)/100 g, non-plant-based: £0.85(0.55)/100 g, U =  69,784.0, *p* < 0.001), pounds per 100 calories (plant-based: £0.53(0.40)/100 kcal, non-plant-based: £0.49(0.42)/100 kcal, *U* =  79,580.5, *p* = 0.019) and pounds per 100 g of protein (plant-based: £8.67(6.25)/100 g protein, £5.46(3.65)/100 g protein, U =  46,467.0, *p* < 0.001) was significantly higher for plant-based products compared with non-plant-based products. These findings were more consistent across product categories (Table 2).

**Table 3.** Nutritional content and cost of plant-based and non-plant-based menu items in UK restaurants, compared using the Wilcoxon Signed Rank test.

	Plant-based				Non-plant-based				
	*n*	missing (%)	Median	IQR	*n*	missing (%)	Median	IQR	*p*-value
Item weight (g)	43	20.4	391.2	418.7	43	20.4	398.0	472.0	**0**.**012**
Energy (kcal/item)	54	0.0	728.5	831.3	54	0.0	832.5	986.3	**<0**.**001**
Energy (kcal/100 g)	43	20.4	223.8	71.1	43	20.4	234.0	81.9	**<0**.**001**
Fat (g/item)	53	1.9	24.2	25.9	54	0.0	30.0	30.8	**<0**.**001^a^**
Fat (g/100 g)	43	20.4	7.6	3.4	43	20.4	9.4	4.5	**<0**.**001**
Saturated fat (g/item)	53	1.9	8.7	22.7	54	0.0	13.0	16.0	**0**.**017**
Saturated fat (g/100 g)	43	20.4	3.2	2.8	43	20.4	3.6	3.2	0.476
Carbohydrate (g/item)	53	1.9	80.0^b^	121.8	54	0.0	80.0^b^	137.7	**<0**.**001**
Carbohydrate (g/100 g)	43	20.4	28.6	9.5	43	20.4	26.0	10.1	**<0**.**001^a^**
Sugar (g/item)	53	1.9	7.8	10.2	54	0.0	8.2	10.7	**0**.**007**
Sugar (g/100 g)	43	20.4	3.0^c^	2.8	43	20.4	2.6^c^	4.7	**0**.**005^a^**
Fibre (g/item)	30	44.4	10.5	10.4	31	42.6	9.0	10.5	**0**.**009^a^**
Fibre (g/100 g)	21	61.1	2.9	1.1	21	61.1	2.2	0.5	**<0**.**001^a^**
Protein (g/item)	53	1.9	23.0	27.9	54	0.0	38.4	45.8	**<0**.**001^a^**
Protein (g/100 g)	43	20.4	6.9	3.1	43	20.4	10.1	4.0	**<0**.**001^a^**
Salt (g/item)	53	1.9	3.6	4.4	54	0.0	3.8	4.8	0.160
Salt (g/100 g)	43	20.4	1.1	0.4	43	20.4	1.0	0.4	0.586
Cost per total item (£)	54	0.0	12.12	12.65	54	0.0	12.24	13.65	0.392
Cost per 100 g product (£)	43	20.4	3.64	1.18	43	20.4	3.38	1.05	**0**.**003^a^**
Cost per 100 kcal (£)	54	0.0	1.52	0.63	54	0.0	1.37	0.60	**<0**.**001^a^**
Cost per 100 g protein (£)	52	3.7	52.50	28.76	54	0.0	30.93	17.65	**<0**.**001^a^**

^a^ Sign test performed on raw data as a symmetrical distribution of differences between pairs for plant-based and non-plant-based menu items was not achieved using transformations (see Supplemental Material 3 for details).

^b^ Note that whilst the median values are the same for plant-based items compared with non-plant-based items, the *p*-value represents a significantly higher carbohydrate content in plant-based items compared with non-plant-based items.

^c^ Note that whilst median values are higher for plant-based items compared with non-plant-based items, the *p*-value represents a significantly lower sugar content in plant-based items compared with non-plant-based items.

Missing (%) refers to the proportion of products that did not have data available for that variable. Note that the medians are only calculated using values for products that have available data both the plant-based and non-plant-based option for each variable.

g: grams; IQR: interquartile range; kcal: kilocalories; n: number of products with available data.

Statistically significant results using Wilcoxon Signed Rank tests are highlighted in bold.

### Restaurant products

#### Product availability

Data were collected for a total of 54 pairs of plant-based and non-plant-based products in UK restaurants.

#### Nutrition

The median and IQR of the nutritional content for plant-based and non-plant-based items, alongside the results of the Wilcoxon Signed Rank tests and Sign test are shown in Table 3, with *Z* values shown in Supplemental Table 6. The proportion of missing data for each variable is also reported (Table 3).

The energy (*z* = −4.38; *p* < 0.001), fat (*z* = −4.67; *p* < 0.001), saturated fat (*z* = −2.37; *p* = 0.017), sugar (*z* = −2.66; *p* = 0.007), and protein (*z* = −6.24 *p* < 0.001) content per plant-based menu item were significantly lower than for non-plant-based products. The carbohydrate (*z* = 4.16, *p* < 0.001) and fibre (*z* = 2.55; *p* = 0.009) contents of plant-based products per menu item were significantly higher than non-plant-based products. There was no significant difference between the salt content of plant-based and non-plant-based restaurant items (*z* = −1.42; *p* = 0.160).

Data on item weight was available for 43 of the 54 pairs of dishes. The total serving weight of plant-based dishes was significantly lower compared with non-plant-based dishes (z = −2.49; *p* = 0.012). To explore whether differences in the nutritional content of plant-based and non-plant-based menu items could be explained by differences in the weight of items served, the nutritional content of plant-based and non-plant-based menu options was also compared per 100 g (Table 3).

The energy (*z* = −3.96, *p* < 0.001), fat (*z* = −3.73, *p* < 0.001), sugar (*z* = −2.75, *p* = 0.005), and protein (*z* = −5.49, *p* < 0.001) content of vegan menu items were significantly lower compared with non-vegan items per 100 g. The carbohydrate (*z* = 3.86, *p* < 0.001), and fibre (z = 3.58, *p* < 0.001) content of vegan menu items were significantly higher compared with non-vegan menu items per 100 g. There was no significant difference between the saturated fat (*z* = 0.72, *p* = 0.476) or salt (*z* = 0.56, *p* = 0.586) content of vegan and non-vegan menu items per 100 g.

#### Cost

The median and IQR of the cost of plant-based and non-plant-based items, alongside the results of the Wilcoxon Signed Rank tests are shown in Table 3.

There was no significant difference between the cost per menu item of plant-based and non-plant-based products (*z* = 0.88, *p* = 0.392). The cost per 100 g product (*z* = 2.88; *p* = 0.003), cost per 100 calories (*z* = 4.76; *p* < 0.001), and cost per 100 g protein (*z* = 5.88; *p* < 0.001) were significantly higher for plant-based products compared with non-plant-based products.

## Discussion

This study compared both the nutritional content and cost of plant-based meat-imitation products and non-plant-based meat products in UK supermarkets and restaurants. Overall, the median nutritional content of plant-based products was 2.8 times higher for carbohydrate, 1.3 times higher for sugar and 7.7 times higher for fibre, compared with non-plant-based products. On the other hand, non-plant-based products were 1.8 times higher in saturated fat and 1.2 times higher in protein, compared with plant-based products. The higher sugar content of plant-based products reflects the greater proportion of energy provided by carbohydrate in these products, and the 0.2 g/100 g difference in sugar content is not clinically significant in the context of the amount of product likely to be consumed by individuals.

These findings are in keeping with previous studies, which also found that plant-based meat alternatives were lower in saturated fat and protein, and higher in carbohydrate, sugar and fibre (Coffey et al., 2023; Petersen and Hirsch, 2023). This study found that the median cost of plant-based products was 20% more expensive per 100 g product compared with non-plant-based products. Comparison of the nutritional content and cost of plant-based and non-plant-based products across specific product categories showed varying trends across different product types.

Meat, fish and poultry are rich sources of protein, and therefore the significantly lower protein content (3.0 g/100 g product across supermarket categories) may have implications for individuals reliant on meat-alternatives as a protein source. The difference was particularly high for coated fish products, where plant-based imitation products were 7.7 g/100 g lower in protein. Therefore, whilst these products may imitate meat and fish, many cannot be seen as an equivalent alternative in terms of protein content.

When considering the health profile of foods, the nutritional profile may not tell the whole story. Ultra-processed ingredients (both plant-based and non-plant-based) may carry a significant adverse risk to health (Lane et al., 2024). Meat-imitation supermarket products often meet the classification of being ultra-processed (Rizzolo-Brime et al., 2023). Therefore, for consumers considering swapping unprocessed meat (e.g., chicken breast) to an ultra-processed plant-based imitation product, the health implications of the ingredient profile, as well as the nutritional content, must be considered.

This study went further to conduct a nutritional and price comparison of plant-based and non-plant-based products available in UK restaurants. Plant-based restaurant products had a more favourable nutritional profile compared with non-plant-based products. They were significantly lower in energy, fat, saturated fat, and sugar and significantly higher in carbohydrate and fibre compared with non-plant-based products. The sugar content in non-plant-based items was on average 0.4 g/item higher which is unlikely to be clinically significant. However, plant-based products were also significantly lower in protein compared with non-plant-based products. The salt content of plant-based and non-plant-based products was similar. Whilst the cost of plant-based and non-plant-based menu items were comparable per serving, plant-based products were significantly more expensive per 100 g, per 100 calories and per 100 g protein.

The findings of this study are somewhat consistent with the previous literature, which also found that plant-based restaurant items were lower in energy, fat, saturated fat and protein, and higher in carbohydrate and fibre (Guess et al., 2023; Kaminski et al., 2024). Whilst this study found that plant-based products were significantly lower in sugar compared with non-plant-based products, Guess et al., 2023 and Kaminski et al., 2024 found that plant-based products were significantly higher in sugar. Furthermore, this study found no significant difference between the salt content of plant-based and non-plant-based restaurant products, whilst the two previous studies found that plant-based options were significantly lower in salt (Guess et al., 2023; Kaminski et al., 2024).

The results may differ due to a variety of reasons. This study matched restaurant products in pairs of highly comparable plant-based and non-plant-based products, whilst Guess et al. did not match products, and Kaminski et al. matched products based on much broader criteria, such as generic meal type categories (Guess et al., 2023; Kaminski et al., 2024). Furthermore, whilst both previous studies included products from UK restaurants, they also included products from Canada, the USA, Australia and Poland, therefore the range of products included is different.

Whilst consumers may use nutritional labelling to support food choices (Ni Mhurchu et al., 2018), cost is also an important consideration in food purchasing behaviour, particularly for individuals with a low income (Steenhuis et al., 2011). Therefore, the higher cost associated with plant-based products may be a barrier to their purchase for some consumers, particularly those aiming to achieve a cost-effective intake of protein, as the plant-based meat alternatives and restaurant options were significantly more expensive per 100 g of protein across the majority of product categories.

### Strengths and limitations

To the best of our knowledge, this is the largest study to compare the cost of a range of exclusively plant-based and non-plant-based meat, poultry and fish imitation products in UK supermarkets and the first to conduct a cost comparison of plant-based and non-plant-based meals in restaurants. This study builds on existing research comparing the nutritional content of plant-based and non-plant-based products in supermarkets and restaurants.

This study assigned supermarket products to homogenous product groups, and matched plant-based and non-plant-based products in restaurants that would be considered by consumers to be close equivalents. This provides a direct comparison of the nutritional and cost implications for consumers wishing to switch to a highly equivalent plant-based product, which is seen as an acceptable meat alternative by consumers (Szenderák et al., 2022). Despite this, some results in the supermarket comparison had relatively high IQRs, indicating variation in that nutrient level for the specified product category. This was particularly true for several of the plant-based categories for saturated fat and sugar, and for the non-plant-based categories for carbohydrate, sugar and fibre. Therefore, whilst this paper can therefore offer general findings, consumers should be mindful of referring to the labels of individual products, due to variation that exists between products of similar formulations. The wide IQRs rereported alongside the restaurant comparison indicate the range of products included in the comparison (starters, main courses, desserts, sides, etc.).

Whilst several studies have investigated ‘plant-based’ products in supermarkets and restaurants, including both vegetarian and vegan products in the ‘plant-based’ category (Brooker et al., 2022; Costa-Catala et al., 2023; Curtain and Grafenauer, 2019; Dunn et al., 2021; Huybers and Roodenburg, 2023; Krobath et al., 2021), this study focused exclusively on products that contained no animal-derived ingredients. This is an important distinction to make, since consumption of dairy may be unacceptable to those following an exclusively plant-based or vegan diet due to ethical or health reasons (North et al., 2021).

However, this study is not without limitations. Whilst this study did not capture the full market share of UK supermarkets and restaurants, an approach was taken to select the supermarkets with the greatest UK market share, and most popular UK restaurants, to maximise the generalisability of the findings. The products included may not be representative of products sold outside of the United Kingdom.

Due to the separation of products into specific categories to facilitate comparison of analogous meat products and plant-based meat-imitation products, some categories of products had few products eligible for inclusion. This was particularly true for plant-based bacon-style products (6 products available) and ham-imitation products (9 products available), which may result in imprecise results for these product categories.

### Future work

Future studies may capture a wider range of products available in supermarkets and restaurants, by including more retailers in the United Kingdom and internationally. Furthermore, given this market is expanding rapidly (Statista, 2024a), repeating this work in the future in the United Kingdom would be beneficial to reassess the products available, their composition, and cost, as this is likely to change with time.

Comparison of the micronutrient content of plant-based and non-plant-based supermarket and restaurant products would be an important next step given plant-based diets may contain lower levels of several micronutrients, including vitamin B12, calcium, iodine and zinc, compared with non-plant-based diets (Bakaloudi et al., 2021; Key et al., 2022; Neufingerl and Eilander, 2021). A study of plant-based meat alternatives in Australia found that only 12% of products were fortified with B12, iron and zinc (Melville et al., 2023), therefore this may indicate a significant shortfall in the nutritional adequacy of plant-based meat alternatives. A key barrier to understanding this further is limited availability of reported micronutrient content on labels of plant-based meat alternatives, because their declaration is not mandatory (Romão et al., 2023). Furthermore, an evaluation of the ingredients list of plant-based meat alternatives is of interest, to better understand the proportion of plant-based meat alternatives that are ultra-processed, and the potential health impacts of preservatives and additives in these foods.

Lastly, a greater understanding of how plant-based supermarket and restaurant products contribute to the wider diet is of interest. This would be helpful to understand whether the nutritional and cost differences in the products investigated in the present study are significant in the context of the wider dietary intake of individuals.

### Public health implications and conclusion

In conclusion, this study compared the nutritional content and cost of meat, poultry and fish products, and their plant-based imitation products, sold in leading UK supermarkets. Plant-based products were significantly higher in carbohydrate, sugar and fibre, and lower in protein and saturated fat, compared with non-plant-based products. Plant-based meat alternatives were significantly more expensive per 100 g of the product, per 100 calories and per 100 g of protein. Other plant-based sources of protein, for example legumes, are significantly lower in price compared with plant-based meat alternatives (Young et al., 2023). Therefore, utilising these in homemade foods may present a more affordable option.

Secondly, this study compared the nutritional content and cost of plant-based and non-plant-based equivalent products sold in UK restaurants. Plant-based restaurant items were lower in energy, fat, saturated fat, and sugar and higher in fibre compared with non-plant-based products. However, plant-based products were also significantly lower in protein compared with non-plant-based products. There was no significant difference between the salt content of plant-based and non-plant-based products. Whilst the cost of plant-based and non-plant-based menu items were comparable per serving, plant-based products were significantly more expensive per 100 g, per 100 calories and per 100 g protein.

It is challenging to draw a blanket conclusion on whether the nutritional profile of plant-based meat alternatives is favourable over non-plant-based products because plant-based products were both higher in nutrients associated with positive health outcomes (fibre), they are also higher in nutrients associated with detrimental health outcomes (sugar) and contain lower levels of saturated fat. Furthermore, the ultra-processed nature of many plant-based meat alternatives may pose an additional risk (Lane et al., 2024; Rizzolo-Brime et al., 2023). However, for consumers aiming to reduce their calorie, saturated fat and sugar intake, or increase their fibre intake, plant-based options may present a good opportunity to do so in the restaurant setting. Furthermore, cost may not be prohibitive of this recommendation, given this study found no significant difference between the cost of plant-based and non-plant-based products, per serving.

## Supplemental Material

sj-docx-1-nah-10.1177_02601060251344449 - Supplemental material for A cross-sectional study of the cost and nutritional content of plant-based meat-imitation products in supermarkets and plant-based products in restaurants in the United Kingdom Supplemental material, sj-docx-1-nah-10.1177_02601060251344449 for A cross-sectional study of the cost and nutritional content of plant-based meat-imitation products in supermarkets and plant-based products in restaurants in the United Kingdom by Grace Monori, Anjum Memon and Gemma Archer in Nutrition and Health

sj-docx-2-nah-10.1177_02601060251344449 - Supplemental material for A cross-sectional study of the cost and nutritional content of plant-based meat-imitation products in supermarkets and plant-based products in restaurants in the United Kingdom Supplemental material, sj-docx-2-nah-10.1177_02601060251344449 for A cross-sectional study of the cost and nutritional content of plant-based meat-imitation products in supermarkets and plant-based products in restaurants in the United Kingdom by Grace Monori, Anjum Memon and Gemma Archer in Nutrition and Health
