# Supplementary material for: A cross-sectional study of the cost and nutritional content of plant-based meat-imitation products in supermarkets and plant-based products in restaurants in the United Kingdom
Source: Nutr Health. 2025 May 27;32(3):979–90. doi: 10.1177/02601060251344449 (PMC13144640; doi:10.1177/02601060251344449)
Supplement: sj-docx-1-nah-10.1177_02601060251344449 - Supplemental material for A cross-sectional study of the cost and nutritional content of plant-based meat-imitation products in supermarkets and plant-based products in restaurants in the United Kingdom [file sj-docx-1-nah-10.1177_02601060251344449.docx]

**Titles of Supplemental Material**

**Supplemental Material 1:** Inclusion and exclusion criteria of supermarket products

**Supplemental Material 2:** STROBE checklist

**Supplemental Material 3:** Details of normality tests

**Supplemental Table 1:** Search terms used to identify products on UK supermarket websites

**Supplemental Table 2:** Specific inclusion and exclusion criteria relevant to each supermarket product category

**Supplemental Table 3:** Conversion factors for weight change from raw to cooked products

**Supplemental Table 4:** Detailed results of Mann Whitney U tests for nutritional content of supermarket products, including mean ranks and U-values

**Supplemental Table 5:** Detailed results of Mann Whitney U tests for cost of supermarket products, including mean ranks and U-values

**Supplemental Table 6:** Detailed results (Z value) of Wilcoxon Signed Rank tests and Sign tests
