# Supplementary material for: A cross-sectional study of the cost and nutritional content of plant-based meat-imitation products in supermarkets and plant-based products in restaurants in the United Kingdom
Source: Nutr Health. 2025 May 27;32(3):979–90. doi: 10.1177/02601060251344449 (PMC13144640; doi:10.1177/02601060251344449)
Supplement: sj-docx-2-nah-10.1177_02601060251344449 - Supplemental material for A cross-sectional study of the cost and nutritional content of plant-based meat-imitation products in supermarkets and plant-based products in restaurants in the United Kingdom [file sj-docx-2-nah-10.1177_02601060251344449.docx]

**Supplemental Material 1: Inclusion and exclusion criteria of supermarket products**

The following products were included, to ensure representation of the breadth of products available to consumers:

- Own-label and branded products
- Premium, budget, organic and standard ranges, as well as ranges marketed as ‘healthier’, e.g. reduced fat or salt
- Halal products

The following products were excluded:

- Products marketed as “free-from”, e.g. gluten free
- Products for which energy content was not available
- Plant-based products that were not meat or fish imitations
- Plant-based products containing any animal-derived ingredients, e.g. egg, dairy

Specific inclusion and exclusion criteria for each product category are shown in Supplemental Table 2.

**Supplemental Material 2: STROBE checklist**

|  |  | Reporting Item | Page Number |
| --- | --- | --- | --- |
| **Title and abstract** |  |  |  |
| Title | [#1a](https://www.goodreports.org/reporting-checklists/strobe-cross-sectional/info/#1a) | Indicate the study’s design with a commonly used term in the title or the abstract | 1 |
| Abstract | [#1b](https://www.goodreports.org/reporting-checklists/strobe-cross-sectional/info/#1b) | Provide in the abstract an informative and balanced summary of what was done and what was found | 2 |
| **Introduction** |  |  |  |
| Background / rationale | [#2](https://www.goodreports.org/reporting-checklists/strobe-cross-sectional/info/#2) | Explain the scientific background and rationale for the investigation being reported | 3 |
| Objectives | [#3](https://www.goodreports.org/reporting-checklists/strobe-cross-sectional/info/#3) | State specific objectives, including any prespecified hypotheses | 4 |
| **Methods** |  |  |  |
| Study design | [#4](https://www.goodreports.org/reporting-checklists/strobe-cross-sectional/info/#4) | Present key elements of study design early in the paper | 4 |
| Setting | [#5](https://www.goodreports.org/reporting-checklists/strobe-cross-sectional/info/#5) | Describe the setting, locations, and relevant dates, including periods of recruitment, exposure, follow-up, and data collection | 4 |
| Eligibility criteria | [#6a](https://www.goodreports.org/reporting-checklists/strobe-cross-sectional/info/#6a) | Give the eligibility criteria, and the sources and methods of selection of participants. | 4 |
|  | [#7](https://www.goodreports.org/reporting-checklists/strobe-cross-sectional/info/#7) | Clearly define all outcomes, exposures, predictors, potential confounders, and effect modifiers. Give diagnostic criteria, if applicable | 5 |
| Data sources / measurement | [#8](https://www.goodreports.org/reporting-checklists/strobe-cross-sectional/info/#8) | For each variable of interest give sources of data and details of methods of assessment (measurement). Describe comparability of assessment methods if there is more than one group. Give information separately for exposed and unexposed groups if applicable. | 5 |
| Bias | [#9](https://www.goodreports.org/reporting-checklists/strobe-cross-sectional/info/#9) | Describe any efforts to address potential sources of bias | 6 |
| Study size | [#10](https://www.goodreports.org/reporting-checklists/strobe-cross-sectional/info/#10) | Explain how the study size was arrived at | 5 |
| Quantitative variables | [#11](https://www.goodreports.org/reporting-checklists/strobe-cross-sectional/info/#11) | Explain how quantitative variables were handled in the analyses. If applicable, describe which groupings were chosen, and why | 6 |
| Statistical methods | [#12a](https://www.goodreports.org/reporting-checklists/strobe-cross-sectional/info/#12a) | Describe all statistical methods, including those used to control for confounding | 7 |
| Statistical methods | [#12b](https://www.goodreports.org/reporting-checklists/strobe-cross-sectional/info/#12b) | Describe any methods used to examine subgroups and interactions | 7 |
| Statistical methods | [#12c](https://www.goodreports.org/reporting-checklists/strobe-cross-sectional/info/#12c) | Explain how missing data were addressed | 7 |
| Statistical methods | [#12d](https://www.goodreports.org/reporting-checklists/strobe-cross-sectional/info/#12d) | If applicable, describe analytical methods taking account of sampling strategy | NA |
| Statistical methods | [#12e](https://www.goodreports.org/reporting-checklists/strobe-cross-sectional/info/#12e) | Describe any sensitivity analyses | NA |
| **Results** |  |  |  |
| Participants | [#13a](https://www.goodreports.org/reporting-checklists/strobe-cross-sectional/info/#13a) | Report numbers of individuals at each stage of study—eg numbers potentially eligible, examined for eligibility, confirmed eligible, included in the study, completing follow-up, and analysed. Give information separately for exposed and unexposed groups if applicable. | 7 |
| Participants | [#13b](https://www.goodreports.org/reporting-checklists/strobe-cross-sectional/info/#13b) | Give reasons for non-participation at each stage | 7 |
| Participants | [#13c](https://www.goodreports.org/reporting-checklists/strobe-cross-sectional/info/#13c) | Consider use of a flow diagram | NA |
| Descriptive data | [#14a](https://www.goodreports.org/reporting-checklists/strobe-cross-sectional/info/#14a) | Give characteristics of study participants (eg demographic, clinical, social) and information on exposures and potential confounders. Give information separately for exposed and unexposed groups if applicable. | NA |
| Descriptive data | [#14b](https://www.goodreports.org/reporting-checklists/strobe-cross-sectional/info/#14b) | Indicate number of participants with missing data for each variable of interest | 7 |
| Outcome data | [#15](https://www.goodreports.org/reporting-checklists/strobe-cross-sectional/info/#15) | Report numbers of outcome events or summary measures. Give information separately for exposed and unexposed groups if applicable. | 7 |
| Main results | [#16a](https://www.goodreports.org/reporting-checklists/strobe-cross-sectional/info/#16a) | Give unadjusted estimates and, if applicable, confounder-adjusted estimates and their precision (eg, 95% confidence interval). Make clear which confounders were adjusted for and why they were included | 7 |
| Main results | [#16b](https://www.goodreports.org/reporting-checklists/strobe-cross-sectional/info/#16b) | Report category boundaries when continuous variables were categorized | NA |
| Main results | [#16c](https://www.goodreports.org/reporting-checklists/strobe-cross-sectional/info/#16c) | If relevant, consider translating estimates of relative risk into absolute risk for a meaningful time period | NA |
| Other analyses | [#17](https://www.goodreports.org/reporting-checklists/strobe-cross-sectional/info/#17) | Report other analyses done—e.g., analyses of subgroups and interactions, and sensitivity analyses | NA |
| **Discussion** |  |  |  |
| Key results | [#18](https://www.goodreports.org/reporting-checklists/strobe-cross-sectional/info/#18) | Summarise key results with reference to study objectives | 9 |
| Limitations | [#19](https://www.goodreports.org/reporting-checklists/strobe-cross-sectional/info/#19) | Discuss limitations of the study, taking into account sources of potential bias or imprecision. Discuss both direction and magnitude of any potential bias. | 11 |
| Interpretation | [#20](https://www.goodreports.org/reporting-checklists/strobe-cross-sectional/info/#20) | Give a cautious overall interpretation considering objectives, limitations, multiplicity of analyses, results from similar studies, and other relevant evidence. | 10 |
| Generalisability | [#21](https://www.goodreports.org/reporting-checklists/strobe-cross-sectional/info/#21) | Discuss the generalisability (external validity) of the study results | 12 |
| **Other Information** |  |  |  |
| Funding | [#22](https://www.goodreports.org/reporting-checklists/strobe-cross-sectional/info/#22) | Give the source of funding and the role of the funders for the present study and, if applicable, for the original study on which the present article is based | Title page |

**Supplemental Material 3: Details of normality tests**

Nutrient contents and cost data for each category were tested for normality by assessing skewness, the Kolmogorov-Smirnov test, the Shapiro-Wilk test (for categories with less than 50 samples), as well as visual methods, including a frequency histogram, Q-Q plot, and boxplot, to check for the distribution of data and any outliers.

The majority of variables were non-parametric, and therefore non-parametric tests have been used in the analyses.

An example of these outputs is shown below, and show the importance of interpreting all of the tests together, rather than relying on one test alone.

| **Table 3.1:** Results of numerical measures of normality for energy content per 100g of non-vegan bacon | | | |
| --- | --- | --- | --- |
|  | Skewness | **Kolmogorov-Smirnov test**  Statistic  (p-value) | ***Shapiro-Wilk test***  *Statistic*  *(p-value)* |
| Bacon (non-vegan) | 0.662 | 0.084 (0.008) | *0.967 (<0.001)* |
| The Kolmogorov-Smirnov test was used in this instance due to the sample size (n=162) | | | |

| 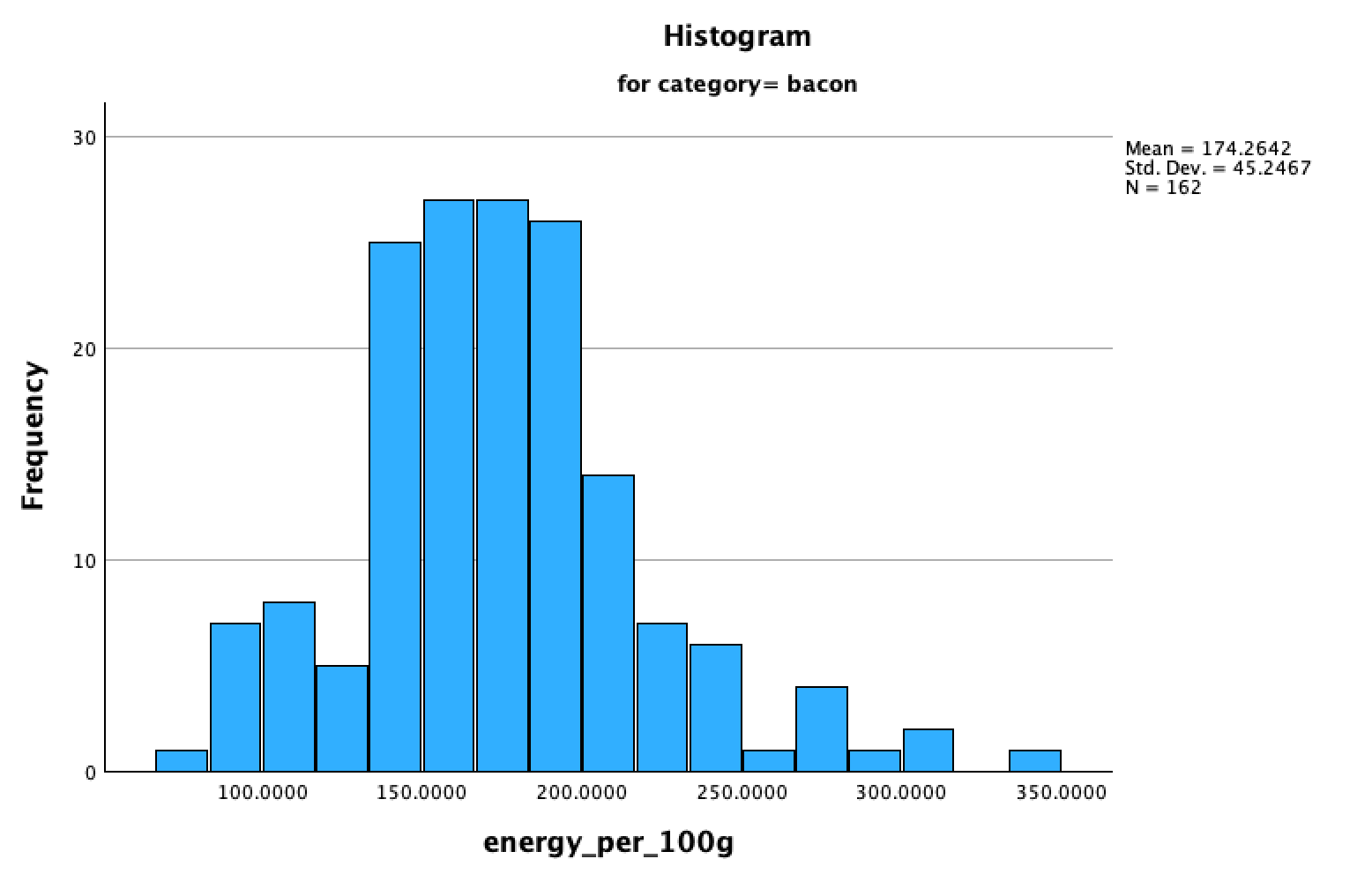 |
| --- |
| **Figure 3.1:** Frequency histogram for energy content per 100g of non-vegan bacon |

| 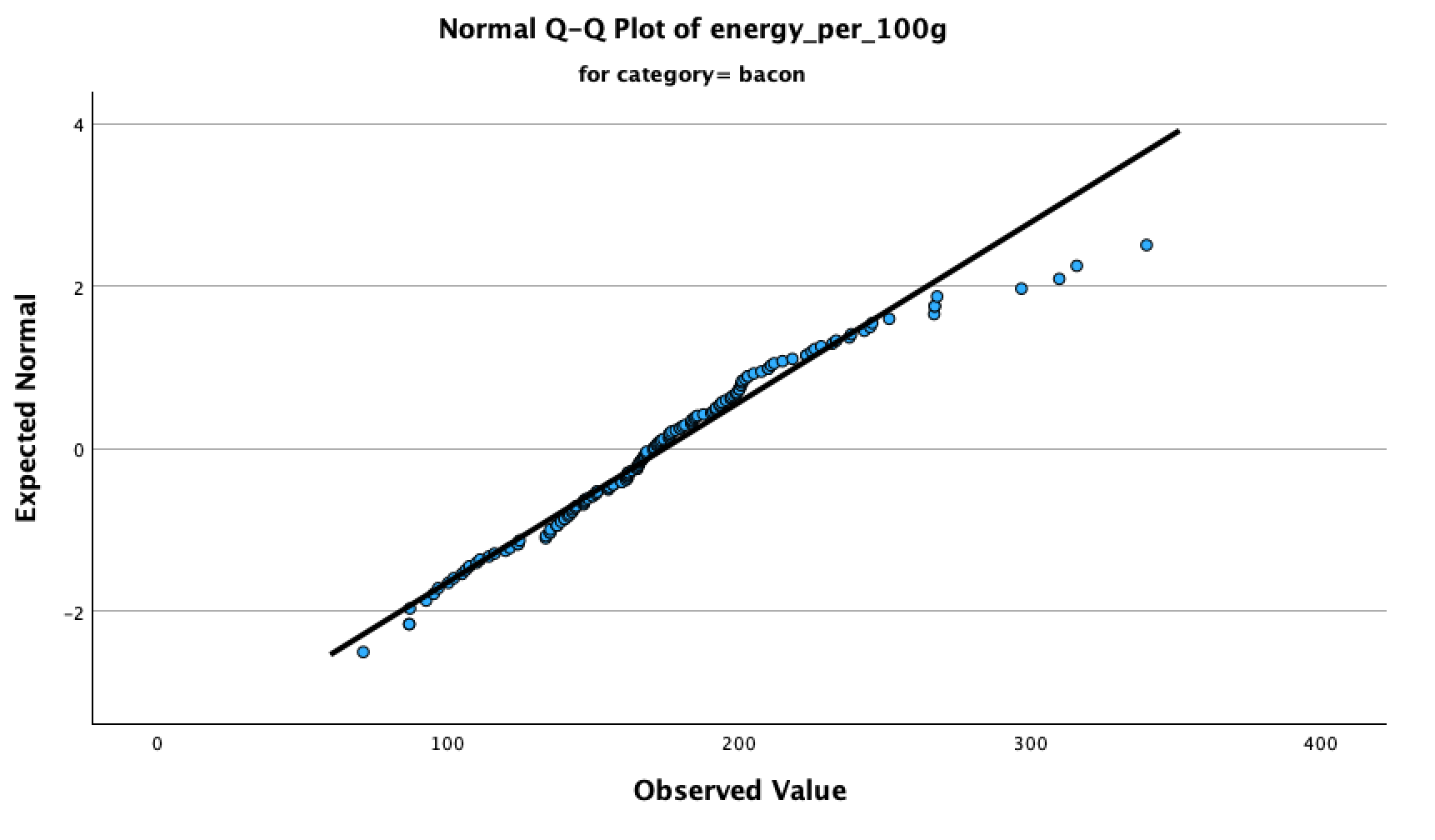 |
| --- |
| **Figure 3.2:** Q-Q plot for energy content per 100g of non-vegan bacon |

| 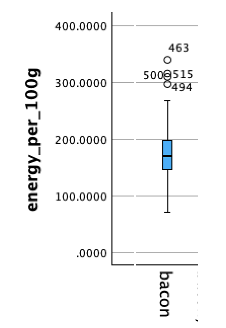 |
| --- |
| **Figure 3.3:** Box plot for energy content per 100g of non-vegan bacon |

**Supplemental Table 1: Search terms used to identify products on UK supermarket websites**

| **Plant-based** | **Non-plant-based** |
| --- | --- |
| Bacon imitation products (fresh)  Search terms: ‘bacon’, ‘medallions’, ‘rashers’ | Bacon (fresh)  Search terms: ‘bacon’, ‘medallions’, ‘rashers’ |
| Beef burger imitation products (fresh/frozen)  Search term: ‘burger’, ‘patties’, ‘quarter pounder’ | Beef burgers (fresh/frozen)  Search term: ‘burger’, ‘patties’, ‘quarter pounder’ |
| Chicken imitation products, coated in batter or breadcrumb (fresh/frozen)  Search term: ‘chicken’ | Chicken, coated in batter or breadcrumb (fresh/frozen)  Search term: ‘chicken’ |
| Chicken imitation products, uncoated (fresh/frozen)  Search term: ‘chicken’ | Chicken, uncoated (fresh/frozen)  Search term: ‘chicken’ |
| Fish imitation products, coated in batter or breadcrumb (fresh/frozen)  Search term: ‘fish’, ‘fishless’ | Fish, coated in batter or breadcrumb (fresh/frozen)  Search term: ‘fish’ |
| Ham slice imitation products (fresh)  Search terms: ‘ham’, ‘slices’ | Ham slices (fresh)  Search term: ‘ham’ |
| Meatball (beef) imitation products (fresh/frozen)  Search terms: ‘meatballs’, ‘Plant-based balls’, ‘plant based balls’ | Meatballs (beef) (fresh/frozen)  Search term: ‘meatballs’ |
| Mince (beef) imitation products (fresh/frozen)  Search term: ‘mince’ | Mince (beef) (fresh/frozen)  Search term: ‘mince’ |
| Sausage/chipolata (pork) imitation products (fresh/frozen)  Search term: ‘sausages’, ‘bangers’, ‘chipolatas’ | Sausages/chipolatas (pork) (fresh/frozen)  Search term: ‘sausages’, ‘bangers’, ‘chipolatas’ |

**Supplemental Table 2: Specific inclusion and exclusion criteria relevant to each supermarket product category**

|  | Inclusion criteria | Exclusion criteria |
| --- | --- | --- |
| Bacon  (fresh) | - Pork products/imitations, e.g. rashers, medallions, streaky bacon - Smoked and unsmoked - Raw, uncooked | - Non-pork products/imitations - Lardons, diced bacon, cooking bacon, gammon steaks, pancetta, cooked bacon, or their imitations |
| Burgers (fresh/frozen) | - Beef products/imitations - Products with added seasoning or ingredients, e.g. jalapenos - Raw, uncooked | - Non-beef products/imitations - Burgers with added cheese or bap - Combination meat and meat alternative products |
| Chicken, coated  (fresh/frozen) | - Any cut of chicken/imitation cut with batter or breadcrumb coating - Boneless, skinless - Any formulation, e.g. burgers, dippers, nuggets, goujons, strips - Raw, uncooked | - Cooked products - Products with a sauce |
| Chicken, uncoated  (fresh/frozen) | - Chicken products/imitation of any cut, without a coating - Boneless, skinless - Raw, uncooked | - Marinated or coated products, burgers, mince, sausages - Products with bones or skin - Cooked products |
| Fish, coated in batter or breadcrumb  (fresh/frozen) | - Boneless white fish/imitation coated in batter or breadcrumb - Any formulation, e.g. fillets, burgers, fish fingers, goujons, bites - Products with added omega 3 - Raw, uncooked | - Non-white fish, or imitations - Fishcakes, pies, fish in hashbrown coating - Products with sauce or ready meals |
| Ham slices  (fresh) | - Pork products/imitations - Smoked, unsmoked - Flavoured, e.g. honey roasted - Seasoned, e.g. peppered | - Non-pork products/imitations - Breaded products or products with fruit coating - Parma ham, pastrami, Serrano ham, prosciutto, ham hock, chorizo, pepperoni, gammon, or their imitations - Products marketed for children, e.g. Paw Patrol slices |
| Meatballs  (fresh/frozen) | - Beef products/imitations, of any fat content - Products with added seasoning/fat - Raw, uncooked | - Non-beef products/imitations - Ready meals containing meatballs, or meatballs in sauce - Cooked products |
| Mince  (fresh/frozen) | - Beef products/imitations, of any fat content - Raw, uncooked | - Non-beef products/imitations - Combination meat and meat alternative products |
| Sausages  (fresh/frozen) | - Pork products/imitations - Added flavours/ingredients, e.g. onion - Raw, uncooked products | - Non-pork products/imitations, e.g. chicken sausages - Black pudding, hot dogs, chorizo, pigs in blankets, or their imitations, or products in batter or pastry - Cooked products |

**Supplemental Table 3: Conversion factors for weight change from raw to cooked products**

| Product | Conversion factor | Number of products applied to | |
| --- | --- | --- | --- |
|  |  | n | %^1^ |
| Bacon, back bacon rashers | 0.68 | 17 | 10.5 |
| Bacon, standard rashers | 0.62 | 2 | 1.2 |
| Bacon, streaky | 0.65 | 6 | 3.7 |
| Burger, beef | 0.66 | 6 | 8.0 |
| Chicken, coated | 0.95 | 18 | 7.9 |
| Chicken, uncoated | 0.75 | 64 | 50.0 |
| Fish, coated | 0.93 | 6 | 3.1 |
| Mince, beef | 0.82 | 7 | 11.3 |
| Sausages, pork | 0.76 | 3 | 1.8 |
| Based on conversion factors published in McCance and Widdowson’s Composition of Foods, 2010 (Food Standards Agency, 2010)  ^1^ Percentage is expressed per number of products in the category, e.g. all bacon products | | | |

***Reference***

Food Standards Agency. (2010) Cooked Foods and Dishes. In *McCance and Widdowson’s The Composition of Foods*. 6th ed. Cambridge: Royal Society of Chemistry.

**Supplemental Table 4: Detailed results of Mann Whitney U tests for nutritional content of supermarket products, including mean ranks and U-values**

|  | **Energy (kcal/100g)** | | **Fat**  **(g/100g)** | | **Saturated fat**  **(g/100g)** | | **Carbohydrate**  **(g/100g)** | | **Sugar**  **(g/100g)** | | **Fibre**  **(g/100g)** | | **Protein**  **(g/100g)** | | **Salt**  **(g/100g)** | |
| --- | --- | --- | --- | --- | --- | --- | --- | --- | --- | --- | --- | --- | --- | --- | --- | --- |
|  | MR | U-value | MR | U-value | MR | U-value | MR | U-value | MR | U-value | MR | U-value | MR | U-value | MR | U-value |
| Bacon |  |  |  |  |  |  |  |  |  |  |  |  |  |  |  |  |
| Plant-based | 93.7 | 431.0 | 68.5 | 390.0 | 14.5 | 66.0 | 162.5 | 0.0 | 158.9 | 39.5 | 161.5 | 0.0 | 45.0 | 249.0 | 71.5 | 408.0 |
| Non-plant-based | 84.2 |  | 85.1 |  | 87.1 |  | 80.0 |  | 81.7 |  | 79.5 |  | 84.4 |  | 85.0 |  |
| Burgers |  |  |  |  |  |  |  |  |  |  |  |  |  |  |  |  |
| Plant-based | 49.4 | 860.5 | 43.4 | 721.0 | 22.9 | 250.5 | 77.0 | 209.5 | 63.7 | 537.0 | 83.0 | 0.0 | 34.2 | 499.0 | 76.8 | 235.0 |
| Non-plant-based | 49.5 |  | 51.4 |  | 57.7 |  | 40.8 |  | 45.2 |  | 36.5 |  | 53.4 |  | 41.1 |  |
| Coated chicken |  |  |  |  |  |  |  |  |  |  |  |  |  |  |  |  |
| Plant-based | 142.5 | 3743.5 | 154.7 | 3305.0 | 112.3 | 3378.0 | 155.4 | 3281.5 | 144.0 | 3689.0 | 216.7 | 351.0 | 56.8 | 1377.0 | 182.4 | 2308.0 |
| Non-plant-based | 130.9 |  | 129.0 |  | 135.7 |  | 128.9 |  | 130.7 |  | 106.2 |  | 144.5 |  | 124.6 |  |
| Uncoated chicken |  |  |  |  |  |  |  |  |  |  |  |  |  |  |  |  |
| Plant-based | 125.8 | 231.0 | 123.1 | 284.0 | 98.9 | 743.0 | 128.9 | 154.0 | 113.7 | 462.5 | 135.0 | 0.0 | 36.2 | 497.0 | 134.7 | 63.0 |
| Non-plant-based | 66.3 |  | 66.7 |  | 70.3 |  | 65.2 |  | 68.1 |  | 63.0 |  | 79.1 |  | 65.0 |  |
| Fish, coated |  |  |  |  |  |  |  |  |  |  |  |  |  |  |  |  |
| Plant-based | 151.7 | 448.5 | 149.6 | 469.5 | 139.4 | 571.5 | 169.1 | 264.0 | 151.4 | 451.0 | 192.5 | 20.0 | 10.4 | 48.5 | 142.6 | 539.0 |
| Non-plant-based | 98.4 |  | 98.5 |  | 99.0 |  | 96.9 |  | 98.4 |  | 95.1 |  | 105.2 |  | 98.8 |  |
| Ham |  |  |  |  |  |  |  |  |  |  |  |  |  |  |  |  |
| Plant-based | 142.3 | 519.5 | 138.9 | 550.0 | 66.7 | 555.0 | 183.4 | 149.0 | 103.4 | 869.0 | 197.0 | 0.0 | 81.2 | 685.5 | 86.0 | 729.0 |
| Non-plant-based | 100.7 |  | 100.8 |  | 104.2 |  | 98.8 |  | 102.5 |  | 96.5 |  | 103.5 |  | 103.3 |  |
| Meatballs |  |  |  |  |  |  |  |  |  |  |  |  |  |  |  |  |
| Plant-based | 13.2 | 81.0 | 12.5 | 71.0 | 7.9 | 12.0 | 25.5 | 6.0 | 24.9 | 14.5 | 24.5 | 0.0 | 9.4 | 31.0 | 23.8 | 29.0 |
| Non-plant-based | 18.7 |  | 19.3 |  | 22.4 |  | 10.3 |  | 10.8 |  | 9.5 |  | 21.4 |  | 11.5 |  |
| Mince |  |  |  |  |  |  |  |  |  |  |  |  |  |  |  |  |
| Plant-based | 30.6 | 251.0 | 24.1 | 185.5 | 16.3 | 108.0 | 67.0 | 0.0 | 63.3 | 42.5 | 67.0 | 0.0 | 14.2 | 82.5 | 66.1 | 14.5 |
| Non-plant-based | 37.5 |  | 38.5 |  | 39.8 |  | 31.5 |  | 32.2 |  | 31.5 |  | 39.2 |  | 31.7 |  |
| Sausages |  |  |  |  |  |  |  |  |  |  |  |  |  |  |  |  |
| Plant-based | 28.2 | 360.5 | 25.7 | 309.5 | 20.3 | 196.0 | 116.1 | 1321.0 | 99.4 | 1671.0 | 173.0 | 0.5 | 76.0 | 1365.5 | 131.8 | 990.5 |
| Non-plant-based | 103.4 |  | 103.7 |  | 104.3 |  | 92.4 |  | 94.5 |  | 81.5 |  | 97.4 |  | 90.4 |  |
| All products |  |  |  |  |  |  |  |  |  |  |  |  |  |  |  |  |
| Plant-based | 738.6 | 82827.0 | 723.4 | 85056.5 | 524.2 | 66172.0 | 888.3 | 59116.5 | 848.2 | 66703.5 | 1243.9 | 2015.5 | 459.3 | 56014.5 | 748.1 | 81419.5 |
| Non-plant-based | 682.0 |  | 683.8 |  | 707.6 |  | 660.3 |  | 668.8 |  | 595.2 |  | 711.2 |  | 680.8 |  |
| Abbreviations – g: grams; kcal: kilocalories; MR: mean ranks.  U-values are a measure of the number of values that rank higher in one group, compared to the comparison group (Laerd Statistics, 2018) | | | | | | | | | | | | | | | | |

**Reference:** Laerd Statistics (2018) *Mann-Whitney U Test using SPSS Statistics*. Available at: <https://statistics.laerd.com/spss-tutorials/mann-whitney-u-test-using-spss-statistics.php> (accessed 19 Jan 2025)

**Supplemental Table 5: Detailed results of Mann Whitney U tests for cost of supermarket products, including mean ranks and U-values**

|  | **Cost per 100g product (£)** | |  | **Cost per 100kcal (£)** | |  | **Cost per 100g protein (£)** | |
| --- | --- | --- | --- | --- | --- | --- | --- | --- |
|  | MR | U-value |  | MR | U-value |  | MR | U-value |
| Bacon |  |  |  |  |  |  |  |  |
| Plant-based | 151.8 | 82.0 |  | 145.5 | 120.0 |  | 159.8 | 16.0 |
| Non-plant-based | 82.0 |  |  | 82.2 |  |  | 80.1 |  |
| Burgers |  |  |  |  |  |  |  |  |
| Plant-based | 66.2 | 478.5 |  | 65.9 | 486.0 |  | 68.2 | 402.0 |
| Non-plant-based | 44.4 |  |  | 44.5 |  |  | 43.4 |  |
| Coated chicken |  |  |  |  |  |  |  |  |
| Plant-based | 181.1 | 2353.5 |  | 172.5 | 2665.5 |  | 218.6 | 1003.0 |
| Non-plant-based | 124.8 |  |  | 126.2 |  |  | 118.9 |  |
| Uncoated chicken |  |  |  |  |  |  |  |  |
| Plant-based | 112.3 | 488.5 |  | 85.5 | 998.0 |  | 121.8 | 288.0 |
| Non-plant-based | 68.3 |  |  | 72.3 |  |  | 66.3 |  |
| Fish, coated |  |  |  |  |  |  |  |  |
| Plant-based | 96.8 | 913.0 |  | 84.4 | 789.0 |  | 182.0 | 135.0 |
| Non-plant-based | 101.2 |  |  | 101.9 |  |  | 96.2 |  |
| Ham |  |  |  |  |  |  |  |  |
| Plant-based | 168.4 | 284.0 |  | 135.1 | 584.0 |  | 186.5 | 121.5 |
| Non-plant-based | 99.5 |  |  | 101.0 |  |  | 98.6 |  |
| Meatballs |  |  |  |  |  |  |  |  |
| Plant-based | 16.2 | 119.0 |  | 17.5 | 111.0 |  | 19.5 | 84.0 |
| Non-plant-based | 16.7 |  |  | 15.8 |  |  | 14.4 |  |
| Mince |  |  |  |  |  |  |  |  |
| Plant-based | 32.9 | 274.0 |  | 32.4 | 269.0 |  | 41.4 | 230.0 |
| Non-plant-based | 37.1 |  |  | 37.2 |  |  | 35.2 |  |
| Sausages |  |  |  |  |  |  |  |  |
| Plant-based | 138.1 | 860.0 |  | 172.1 | 146.0 |  | 152.5 | 556.0 |
| Non-plant-based | 89.6 |  |  | 85.4 |  |  | 87.8 |  |
| All products |  |  |  |  |  |  |  |  |
| Plant-based | 827.3 | 69784.0 |  | 760.6 | 79580.5 |  | 975.5 | 46467.0 |
| Non-plant-based | 671.3 |  |  | 679.3 |  |  | 650.0 |  |
| Abbreviations – g: grams; kcal: kilocalories; MR: mean ranks  U-values are a measure of the number of values that rank higher in one group, compared to the comparison group (Laerd Statistics, 2018) | | | | | | | | |

**Reference:** Laerd Statistics (2018) *Mann-Whitney U Test using SPSS Statistics*. Available at: https://statistics.laerd.com/spss-tutorials/mann-whitney-u-test-using-spss-statistics.php (accessed 19 Jan 2025)

**Supplemental Table 6:** Detailed results (Z value) of Wilcoxon Signed Rank tests and Sign tests

|  | **Z value** |
| --- | --- |
| Item weight (g) | -2.49 |
| Energy (kcal/item) | -4.38 |
| Energy (kcal/100g) | -3.96 |
| Fat (g/item)^a^ | -4.67 |
| Fat (g/100g) | -3.73 |
| Saturated fat (g/item) | -2.37 |
| Saturated fat (g/100g) | 0.72 |
| Carbohydrate (g/item) | 4.16 |
| Carbohydrate (g/100g)^a^ | 3.86 |
| Sugar (g/item) | -2.66 |
| Sugar (g/100g)^a^ | -2.75 |
| Fibre (g/item)^a^ | 2.55 |
| Fibre (g/100g)^a^ | 3.58 |
| Protein (g/item)^a^ | -6.24 |
| Protein (g/100g)^a^ | -5.49 |
| Salt (g/item) | -1.42 |
| Salt (g/100g) | 0.56 |
| Cost per total item (£) | 0.88 |
| Cost per 100g product (£)^a^ | 2.88 |
| Cost per 100kcal (£)^a^ | 4.76 |
| Cost per 100g protein (£)^a^ | 5.88 |
| ^a^ A Sign test was performed for these variables, with a Wilcoxon Signed Rank test performed for the other variables  The Z value represents how many standard deviations the observed sum of signed ranks deviates from its expected value under the null hypothesis. | |
